# The *Cebpd* (C/EBPδ) Gene Is Induced by Luteinizing Hormones in Ovarian Theca and Interstitial Cells But Is Not Essential for Mouse Ovary Function

**DOI:** 10.1371/journal.pone.0001334

**Published:** 2007-12-19

**Authors:** A-Mei Huang, Martina Rudelius, Shikha Sharan, Jan M. McAllister, Mark Raffeld, Lane K. Christenson, Esta Sterneck

**Affiliations:** 1 Center for Cancer Research, National Cancer Institute, Frederick, Maryland, United States of America; 2 Laboratory of Pathology, National Cancer Institute, Bethesda, Maryland, United States of America; 3 Department of Cellular and Molecular Physiology, Pennsylvania State University College of Medicine, Hershey, Pennsylvania, United States of America; 4 Department of Molecular and Integrative Physiology, University of Kansas Medical Center, Kansas City, Kansas, United States of America; Texas Tech University Health Sciences Center, United States of America

## Abstract

The CCAAT/enhancer binding protein (CEBP) family of transcription factors includes five genes. In the ovary, both *Cebpa* and *Cebpb* are essential for granulosa cell function. In this study we have explored the role of the *Cebpd* gene in ovarian physiology by expression and functional studies. Here we report that *Cebpd* (C/EBPδ) is expressed in the mouse ovary in a highly restricted temporal and spatial pattern. In response to luteinizing hormone (LH/hCG), CEBPD expression is transiently induced in interstitial cells and in theca cells of follicles from the primary to pre-ovulatory stage, and overlaps in part with expression of the alpha-smooth muscle actin protein. Efficient down-regulation of CEBPD was dependent on a functional *Cebpb* gene. Proliferating human theca cells in culture also express *Cebpd*. Cells from patients with polycystic ovarian syndrome (PCOS) exhibited higher *Cebpd* expression levels. However, deletion of *Cebpd* in mice had no overt effect on ovarian physiology and reproductive function. Very little is known at present about the molecular mechanisms underlying theca/interstitial cell functions. The expression pattern of CEBPD reported here identifies a novel functional unit of mouse theca cells of primary through tertiary follicles responding to LH/hCG together with a subset of interstitial cells. This acute stimulation of CEBPD expression may be exploited to further characterize the hormonal regulation and function of theca and interstitial cells.

## Introduction

The ovary is a complex organ undergoing regular phases of re-organization in response to a variety of intrinsic and extrinsic factors. The main functional unit, the follicle is comprised of a central oocyte, mural and cumulus granulosa cell layers, and an outer layer of theca cells. Ovarian follicles reside within the interstitial stroma, which also gives rise to the specialized follicular cell types. The ovulatory surge of luteinizing hormone (LH), produced by the pituitary, triggers a series of dramatic morphological and physiological changes in the pre-ovulatory follicle, culminating in ovulation and subsequent differentiation of the follicular granulosa and theca cells into the luteal cells. While the LH receptor signals primarily through the cAMP/PKA pathway to influence gene expression [1] it also impacts signaling pathways involving Janus and phosphoinositol kinases, chloride currents, and calcium [2]–[5]. Transcription factors known to act downstream of LH receptor activation include STAT1 and STAT5 [2], GATA4 [6], EGR1 [7], and the CCAAT/enhancer binding protein proteins CEBPA and CEBPB [8].

The CEBP family of transcription factors is comprised of five proteins with a highly homologous carboxyterminal leucine-zipper/basic region domain required for dimerization and DNA binding. Each CEBP protein has unique properties regulating cell type-specific growth and differentiation. For example, within the hematopoietic system CEBPA is required for development of granulocytes, while lack of CEBPB affects differentiation of the B-cell lineage and monocytes. During adipocyte differentiation, CEBPB, -D, and A are expressed consecutively [9]. In the mammary gland, CEBPB promotes proliferation and differentiation [10], while CEBPD participates in the initiation of cell death [11]. In the ovary, both CEBPA and CEBPB are expressed in follicular granulosa cells, dynamically regulated by gonadotropins, and essential for follicular development, efficient ovulation and luteal differentiation. In particular, CEBPB is a downstream effector of the LH signaling pathway in granulosa cells [8]. The absence of significant levels of CEBPA and CEBPB in theca cells combined with the importance of these factors in mediating LH-triggered events in granulosa cells suggested the possibility that another member of the family may substitute for their function in theca cells. Therefore, we investigated the expression and function of CEBPD in the ovary.

*Cebpd* (C/EBPdelta, CELF, CRP3, NFIL-6beta) was first characterized as an acute phase inflammatory response gene. Expression of *Cebpd* is typically low to undetectable in most cell types and tissues, but is rapidly induced by a variety of extracellular stimuli, (e.g. growth hormone, insulin, IFNgamma, IL-1, IL-6, LPS, TNFalpha, noradrenaline and glutamate) [9], [12]. *In vitro* and *in vivo* studies have implicated CEBPD in proliferation of osteoblasts [13], [14], differentiation of lung epithelial cells [15]–[17], and growth arrest of mouse mammary epithelial cells [18]. While *Cebpd*-deficient mice display no overt phenotype, are fertile and achieve normal life spans, more detailed characterization revealed that the null mutation led to improved performance in the contextual fear conditioning test of long term memory [19], increased mammary ductal branching [20] and delayed mammary gland involution [11]. Furthermore, *Cebpd*-deficiency exacerbates the differentiation defect of *Cebpb*-deficient adipocytes [21], and causes genomic instability in fibroblasts [22]. Thus, CEBPD appears to have highly diverse functions depending on cell type and specific physiological stimuli. Furthermore, because its expression is mostly activated by transient signals, the role of CEBPD *in vivo* may be modulatory and only uncovered when cells are investigated in response to specific stimuli. In this study we have addressed whether CEBPD plays a role in ovarian physiology through expression analyses and a thorough reproductive characterization of *Cebpd*-deficient mice.

## Results

### Dynamic regulation of Cebpd mRNA expression in the mouse ovary

**Figure 1:**
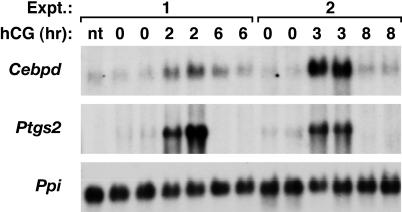
*Cebpd* mRNA expression is induced by hCG in mouse ovaries. Northern blot analysis of whole ovary total RNA isolated from 5 week-old (Exp.1) or 3 week-old (Exp.2) mice treated for 2 days with PMSG followed by the LH analog hCG for the indicated times (n.t. = no treatment). *Ptgs2* was used as a positive control for the hCG response. Cyclophilin (*Ppi*) was used as loading control.

To address the role of CEBPD in the mouse ovary we first assessed its expression pattern in response to LH administration. We used an ovulation protocol in which mice were treated with pregnant mare serum gonadotropin (PMSG) to stimulate the coordinated development of multiple pre-ovulatory follicles, followed two days later by human chorionic gonadotropin (hCG) to mimic the ovulatory surge of LH. Northern blot analysis of whole ovary RNA revealed a highly restricted temporal expression pattern for *Cebpd* (Fig. 1). Expression of *Cebpd* mRNA was minimal prior to hCG treatment, but was highly induced 2–3 hours following hCG, before returning to pre-hCG levels by 6 hours where it remained through 8 hours. Similar data were obtained by Western analysis of whole ovary protein extracts (data not shown). Expression of prostaglandin-endoperoxide synthase 2 (*Ptgs2*, a.k.a. Cox-2, Pghs 2), a gene known to be induced by LH in granulosa cells [23], was evaluated as a positive control (Fig. 1). As expected, ovarian *Ptgs2* expression increased following hCG treatment and then declined rapidly. In whole ovary extracts, *Cebpd* and *Ptgs2* mRNA expression were regulated temporally in a similar pattern (Fig. 1). Similar expression data were obtained from 3 week old prepubertal and 5 week old postpubertal mice. Thus, we have used 5–6 week old mice for all other expression analyses in this study.

### Analysis of *Cebpd* expression in *Cebpb* null mice

**Figure 2 pone-0001334-g002:**
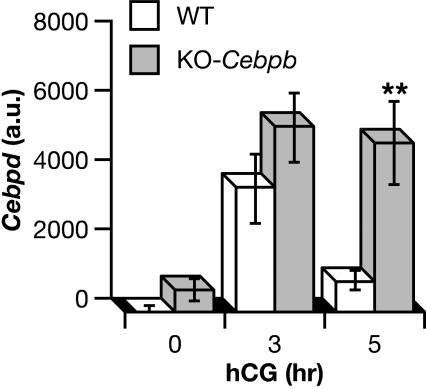
Down-regulation of *Cebpd* mRNA requires *Cebpb*. Phosphorimage quantitation of Northern blot analyses of whole ovary total RNA from 5 week-old *Cebpb* null mice (KO) or litter mate controls (WT) treated for 2 days with PMSG followed by hCG for the indicated times. Expression levels of *Cebpd* were normalized to cyclophilin (*Ppi*) expression and are shown in arbitrary units (a.u.; mean±SEM; n = 3–4 mice per data point). **P = 0.01 by two-way ANOVA with follow-up comparisons among groups in the form of *t*-tests at each time point.

Since we previously identified an essential role of *Cebpb* in the LH responsiveness of granulosa cells [24], we assessed *Cebpd*-expression also in *Cebpb*-deficient mice (Fig. 2). *Cebpd* mRNA expression was induced normally by hCG in *Cebpb* null mice. Expression levels at 0 h and 3 h after hCG treatment were statistically similar in WT and KO ovaries. However, *Cebpd* mRNA levels remained high in *Cebpb* knockout mice through five hours of hCG treatment, in contrast to the rapid loss seen in the controls (Fig. 2). By 8 hours following hCG, *Cebpb*-deficient and control ovaries expressed similar levels of *Cebpd* mRNA (data not shown). This pattern of normal induction by hCG but delayed down-regulation is similar to the defective down-regulation of *Ptgs2* and aromatase mRNA expression in granulosa cells of *Cebpb*-deficient mice [24].

### Localization of *Cebpd* expression in theca/interstitial cells

**Figure 3:**
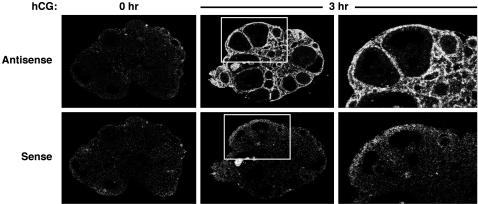
*Cebpd* mRNA expression is localized to theca and interstitial cells. Dark-field photomicrographs of *in situ* hybridization analyses using *Cebpd*-specific sense and antisense RNA probes on sections from ovaries of 6 week-old mice treated for 2 days with PMSG followed by hCG for the indicated times. Outlined regions in the center panels are shown at higher magnification on the right.

**Figure 4:**
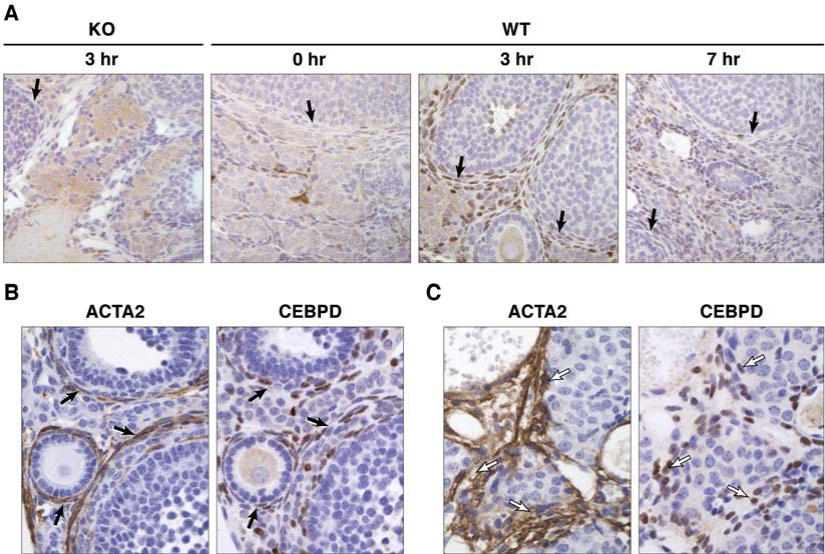
CEBPD protein expression in mouse ovaries. (A) Immunohistochemistry for CEBPD in ovaries from wild-type (WT) or *Cebpd* null mutant (KO) mice at the indicated time points following hCG treatment. (B, C) Immunohistochemistry for CEBPD and alpha smooth muscle actin (ACTA2) in wild-type ovaries harvested 3 h after hCG treatment. Solid arrows indicate theca layers (B), open arrows indicate stromal areas (C) expressing both CEBPD and ACTA2.

To determine which ovarian cell type(s) *Cebpd* was expressed in we employed *in situ* hybridization and immunohistochemistry. Figure 3 confirms the relative lack of *Cebpd* expression prior to the LH surge, and the dramatic induction of *Cebpd* mRNA levels following hCG administration. This expression was localized in the theca cell layer of follicles of varying sizes as well as in the stromal compartment. No evidence of granulosa cell expression was observed in any preparation. To better characterize *Cebpd* expression within the stroma, immunohistochemistry was performed. The anti-CEBPD rabbit polyclonal antibody detected nuclear CEBPD protein expression in theca and interstitial cells specifically in wild-type ovarian tissue but not in *Cebpd* knockout control tissue (Fig. 4A). As seen with *Cebpd* mRNA, maximal protein levels were observed 3 h after hCG treatment. However, 7 h following hCG-adminstration to mice CEBPD protein was still detected in interstitial cells, but less so in theca cells (Fig. 4A). Thus, immunohistochemistry revealed expression kinetics that were not evident by analysis of whole ovary mRNA (Fig. 1) or protein extracts (data not shown). Most of the CEBPD-staining interstitial cells exhibit small, elongated nuclei. Theca externa cells express alpha smooth muscle actin (ACTA2) [25], and CEBPD is also expressed in vascular smooth muscle cells [26]. Thus, we sought to determine if CEBPD characterizes ACTA2 expressing interstitial cells. Analysis of parallel sections revealed that ACTA2 and CEBPD stain the same layers in primary to antral follicles (Fig. 4B). Similarly, some stromal areas that stain positive for ACTA2 also contain CEBPD-expressing cells (Fig. 4C).

### Analysis of Cebpd null mutant mice

**Figure 5:**
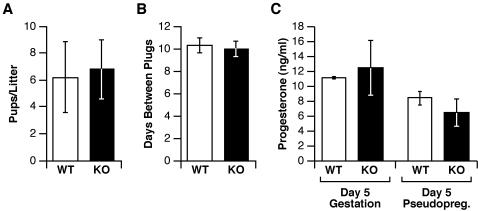
*Cebpd*-deficient females exhibit normal reproductive functions (A) *Cebpd*-deficient females generate normal-sized litters. Two to three-month old females with wild-type *Cebpd* (WT, n = 10) or *Cebpd* null mutation (KO, n = 16) were mated, the male removed subsequent to recording of a vaginal plug, and the number of pups of the following litter were counted within 24 hours after birth. (B) *Cebpd*-deficient females display regular estrous cycles. Two month-old *Cebpd* null females (KO, n = 5) and wild-type littermate controls (WT, n = 4) were mated to vasectomized, sterile males and monitored daily for the occurrence of vaginal plugs over a period of 40–50 days. The number of days between 19 plugs per group was averaged. (C) *Cebpd*-deficient females display normal progesterone levels. Progesterone levels were measured in serum from mice on day 5 of gestation (from experiment in Fig. 5A, n = 5 KO, 3 WT) or on day 5 of pseudopregnancy (from experiment in Fig. 5B, n = 5 KO, 4 WT). Means±S.E.M. are shown.

When *Cebpd* null mutant mice had first been generated [19], females and males were found to be fertile. Here we conducted a more detailed analysis to potentially uncover effects of *Cebpd* deficiency on ovarian physiology that may not be evident in assays of fertility. Comparison of *Cebpd* and control mice reproductive parameters are shown in Figure 5. No differences were observed between *Cebpd* knockout and control mice with respect to the size of their first litters (Fig. 5A). Regulation of the estrous cycle was assessed indirectly by mating the females to sterile males and recording mating activity. Both genotypes mated on average every ten days, indicating normal mating behavior and a normal pseudopregnancy response to mating, which is reliant on luteal progesterone production (Fig. 5B). Levels of progesterone were assessed as a measure of follicular differentiation following ovulation. *Cebpd* null mice and controls produced similar levels of progesterone on day 5 of gestation and day 5 of pseudopregnancy (Fig. 5C). Lastly, examination of the ovarian histology of mice from age 70 to 400 days did not reveal abnormalities when compared to wild-type littermates (data not shown). Taken together, these data demonstrate that the *Cebpd* null mutation does not overtly affect reproductive function in female mice.

**Figure 6:**
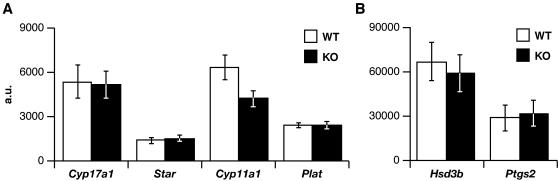
Normal gene expression pattern in *Cebpd*-deficient ovaries. Phosphorimage quantification of Northern blot analyses for the indicated genes of whole ovary total RNA from 5 week-old *Cebpd* null mice (KO) and litter mate controls (WT) treated for 2 days with PMSG followed by hCG for 4 hours. Expression levels were normalized to cyclophilin (*Ppi*) expression (a.u. = arbitrary units; means±SEM). Panel A: n = 6 WT, 8 KO; Panel B: n = 4 WT, 4 KO.

Since a physiological response may proceed normally despite the altered regulation of certain genes, we also analyzed the expression of several marker genes 4 hours after hCG treatment in knockout and wild-type ovaries (Fig. 6). No differences were observed for the mRNA levels of several steroidogenic marker genes, steroidogenic acute regulatory protein (*Star*), cytochrome P450scc (*Cyp11a1*), 17-α hydroxylase (*Cyp17a1*), and 3β-hydroxysteroid dehydrogenase (*Hsd3b*), as well as the theca-specific tissue-type plasminogen activator (*Plat*) gene [27]. Furthermore, *Ptgs2*, a gene primarily expressed in granulosa cells [27], was induced to similar levels in *Cebpd* knockout and control mice (Fig. 6). These data further confirm normal ovarian function in the absence of CEBPD.

### Expression of *CEBPD* in cultured human theca cells

**Figure 7:**
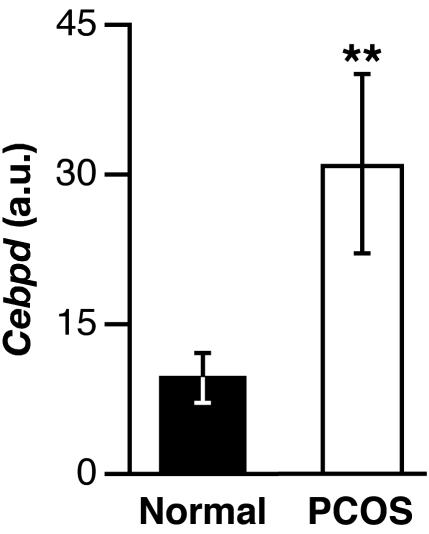
*Cebpd* is overexpressed in theca cells from PCOS patients. Quantitative real-time RT-PCR results examining *Cebpd* mRNA expression in theca cells derived from four healthy women and four PCOS patients are shown. Expression levels were normalized by *Gapdh* values and the relative values are shown (a.u. = arbitrary units). The two-sample independent *t*-test, for which the values were transformed to their common logs, and the nonparametric Mann-Whitney test, both yielded a statistically significant difference at *P*<0.03.

Analysis of cultured proliferating theca cells obtained from patients with polycystic ovarian syndrome (PCOS, n = 4) showed approximately 3-fold higher levels of *Cebpd* mRNA than theca cells isolated from women not afflicted with this syndrome (Fig. 7). These data confirm that *Cebpd* expression in theca cells is not a species-specific phenomenon as it occurs in mouse and human. Previous microarray experiments utilizing the same RNA preparations used in our experiments showed increased 17α-hydroxylase (*Cyp17a1*) expression in these PCOS-theca cells [28], confirming their pathological condition.

## Discussion

The CEBP family of transcription factors has many different functions in a variety of physiological processes. In the ovary, CEBPA and CEBPB play key roles in the LH response of the follicle. *Cebpa* is widely and constitutively expressed in the rat ovary. *Cebpa* expression levels do not change dramatically upon hormone stimulation, and localize mostly to the granulosa cells and theca cells of preovulatory follicles, with the highest levels in cumulus cells. On the other hand *Cebpb* expression is rapidly and highly induced by hCG mainly in granulosa cells. Knockdown and targeted mutations have shown that both CEBPA and CEBPB are important for ovulation and *Cebpb*-deficient mice are sterile presumably due to a granulosa cell defect [8]. We used a combination of expression studies and genetic analysis to assess the function of CEBPD in the mouse ovary. We found that in contrast to CEBPA and CEBPB, the CEBPD protein does not have a critical or non-redundant role in the ovary. However, expression of the *Cebpd* gene is acutely responsive to the LH surge in a select group of interstitial cells within the ovarian stroma and in theca cells of follicles. The diversity of cell type and hormone-response specific expression of different *Cebp* genes demonstrates that this gene family has been exploited in evolution to contribute to the development of the ovary by providing unique functions for its variety of cell types. Interestingly, expression of CEBPD in theca cells is not restricted to pre-ovulatory follicles but was seen at all stages of follicular development, beginning with primary follicles. In the stroma, we observed that most alpha smooth muscle actin containing regions harbor CEBPD-expressing cells, and were thus identified as early responders to LH/hCG. To our knowledge, the only other transcription factor gene previously reported as activated by LH/hCG specifically in theca cells, is the zinc finger protein gonadotropin-inducible transcription factor 1 (*Giot1*). However, activation of Giot1 was detected only in theca interna cells and was not observed in interstitial cells [29]. Remarkably, loss of CEBPD did not have a detrimental effect on thecal cell function, as might be expected, if it was essential for LH-induced steroidogenesis (which is critical for follicular steroidogenesis) or thecal cell development. However, it is possible that loss of CEBPD was compensated by one of the other CEBPs. For example, *Cebpe* mRNA is detectable in whole ovary RNA, but its hormonal regulation or cellular distribution has not yet been characterized [8].

A role for CEBPB in down regulation of *Cebpd* mRNA levels was also observed after its initial activation by hCG. *Cebpb* is mainly expressed in the inner follicular mass of granulosa cells. While we cannot rule out a direct effect of low levels of CEBPB within theca/interstitial cells as the primary mechanism for the observed decline in *Cebpd* mRNA levels, it is tempting to speculate that thecal *Cebpd* expression is regulated by juxtacrine/endocrine factors from the granulosa cell compartment. A number of cytokines, including several members of the EGF family, which are induced in the granulosa layer by LH [27], [30], are dramatically downregulated in *Cebpb* null ovaries (L.K.C., E.S., unpublished data) and, therefore, represent candidate feedback signals.

The observation that the *Cebpd* gene is not essential for fertility in mice, does not rule out that the protein participates in ovarian gene regulation. Therefore, misexpression or overactivity of CEBPD could impact ovarian function in adverse ways. In humans, hyperactivity of the theca/interstitial compartment is associated with PCOS. In the present study, we evaluated *Cebpd* levels in PCOS patient derived theca cells. Our results indicate that *Cebpd* expression was elevated in theca cells that are producing androgen and are in agreement with the chronically elevated androgen levels and *Cyp17a1* gene expression seen in PCOS theca cells. However, our data are in contrast to an array-based expression analysis of biopsies, which reports 2-fold lower levels of *Cebpd* expression in PCOS tissue [31]. There are several potential reasons for this discrepancy. The cultured cells used here were derived from the theca layer of 5–6 mm follicles of both normal and PCOS women, while the biopsies were random and therefore of varying cell type composition that could be quite heterogeneous. In contrast, the cultured theca cells from normal and PCOS women would be more homogenous and comparable cell populations, and cultured cells are known to maintain some of their *in vivo* characteristics unique to PCOS such as elevated *Cyp17a1*. However, the potential effect of culture conditions affecting gene expression patterns can not be discounted either. Because of the relatively small number of samples in this analysis we primarily conclude that *Cebpd* expression in theca cells is not specific to mice but also true for human cells. Our present results lend support but far from confirm that CEBPD is possibly elevated in thecal cells of PCOS women.

In summary, our data show that the *Cebpd* gene is rapidly induced by LH/hCG in theca cells of primary follicles through pre-ovulatory follicles as well as in interstitial cells. This observation demonstrates some common characteristics in the hormonal regulation between these cell populations. The data also suggest that in contrast to CEBPA and CEBPB, CEBPD function is not directly related to the ovulation process, but may rather have a role in the broad endocrine activity of the theca/interstitial compartment, or possibly the inflammatory response associated with ovulation. The physiologic/developmental role of LH/hCG responsive cells within the ovarian stroma compartment is presently not understood. CEBPD can be a useful marker for the early response of the theca/interstitial compartment to LH/hCG and may help to further characterize these ovarian cell types.

## Materials and Methods

### Mice

The generation of mice (*Mus musculus*) with targeted deletion of *Cebpd* [19] and *Cebpb* [24] has been described previously. Wild-type and mutant subjects were offspring from heterozygous breeding pairs. *Cebpb*-deficient mice are not viable on a pure genetic background. Thus, the expression data in Figures 1–3 were derived from mice of a 129B6-F1 strain background, generated by mating heterozygous mice that had been backcrossed into the 129S1 and C57BL/6 strain background, respectively. All other data were generated with mice on a pure 129S1 strain background, which exhibit similar *Cebpd* expression as the 129B6-F1 strain. Where indicated, mice were given intraperitoneal injections of pregnant mare serum gonadotropin (PMSG, 5 IU, SIGMA; equivalent to equine chorionic gonadotropines, eCG) or human chorionic gonadotropin (hCG, 5 IU, SIGMA). The mice were housed and bred in a specific pathogen-free facility with a 12 hour light cycle, and with chow and water *ad libitum*. All procedures were conducted in compliance with the guidelines of the Animal Care and Use Committee of the National Cancer Institute, MD, U.S.A.

### In situ analysis

Ovaries were fixed in 4% paraformaldehyde and 5 µm sections were prepared from paraffin embedded tissues. *In situ* hybridization analysis was performed as described [32], using a cRNA probe representing the coding region for amino acids 1–181 of *Cebpd*.

### Immunohistochemistry

Immunohistochemical staining was performed with the aid of an automated immunostainer (DakoCytomation, Carpinteria, CA). Formalin-fixed paraffin embedded tissue sections were mounted on glass slides and deparaffinized. Prior to staining, heat-induced antigen retrieval was performed by placing the slides into target retrieval solution, high pH (DakoCytomation), and steaming them in a commercial vegetable steamer at full temperature for 30 minutes (CEBPD) or by pressure cooking in TRIS/Citrate (pH 6) for 8 minutes (alpha-smooth muscle actin, ACTA2). Following the antigen retrieval procedure the slides were incubated with a CEBPD rabbit polyclonal antibody (ActiveMotif cat#39006; dilution 1∶500) overnight at 4°C, or a monoclonal mouse antibody against ACTA2 (DAKO, Clone 1A4, dilution 1∶500) for 1 hour. Detection was carried out on the automated system using an HRP/DAB polymer based rabbit detection system (Envision+, DakoCytomation) according to the manufacturer's recommendations.

### Progesterone assay

Serum was prepared from orbital eye bleeds and progesterone levels were assessed with the DSL-10-3900 ACTIVE progesterone EIA kit (Diagnostic Systems Laboratories, Inc.), according to the manufacturer's instructions. Each sample was assayed in duplicate. The coefficient of variation across experiments and genotypes was 0.35.

### RNA analysis

Total ovarian RNA was prepared by homogenization of tissue in Trizol reagent (Life Technologies, Inc.) according to the manufacturer's protocol. The RNA was analyzed by Northern blotting as described [33] except that hybridizations were carried out in HybPlus solution (SigmaAldrich, Inc.). Radiolabeled DNA probes were prepared from isolated mouse cDNA clones for the indicated genes. The specific signals were recorded and quantified by phosphorimage analysis (Molecular Dynamics, ImageQuant™).

### Human theca cell culture and real time PCR analysis

Human theca interna tissue was obtained from follicles of women undergoing hysterectomy, following informed consent under a protocol approved by the Institutional Review Board of the Pennsylvania State University College of Medicine. In the experiments presented in this manuscript, 3^rd^ and 4^th^ passage (31–38 population doublings) theca cells isolated from size-matched follicles obtained from age-matched subjects were used as previously described [34], [35]. All of the PCOS theca preparations studied came from ovaries of women with fewer than six menstrual periods per year and elevated serum total testosterone (T) or bioavailable T levels, as we previously described (26). The control (normal) theca cell preparations came from ovaries of fertile women with normal menstrual histories, menstrual cycles of 21–35 d, and no signs of hyperandrogenemia. The OB/Gyn department at Penn State Medical Center routinely schedules hysterectomies of reproductive aged women during their follicular phase.

Total RNA (5 µg) isolated with Trizol reagent (Life Technologies, Inc.) was treated with DNase I (Promega, Madison, WI) followed by cDNA synthesis using the MMLV reverse transcriptase (Promega) and oligo dT primer (Promega) as previously described [35]. The resulting cDNAs were diluted 1∶100 in sterile water and 1-µl aliquots were used in the quantitative real-time PCRs reactions. The primers used to quantify *Cebpd* (Forward: 5′-GGTGCCCGCTGCAGTTT-3′; Reverse: 5′-CTCGCAGTTTAGTGGTGGTAAGTC-3′) were designed with Primer Express software package that accompanies the Applied Biosystems Model 7900 sequence detector (PerkinElmer Life Science). The SyBr green reagent PCR Master Mix (Applied Biosystems) was used as previously described [36]. In order to account for differences in starting material, the human glyceraldehyde-3-phosphate dehydrogenase (*GAPDH*) primers and probe reagents from Applied Biosystems were used as described by the manufacturer. In order to quantify differences, the samples were compared to standard curves for each target amplicon and the average value for the triplicate was used in all subsequent calculations.
